# 8q24 amplified segments involve novel fusion genes between *NSMCE2* and long noncoding RNAs in acute myelogenous leukemia

**DOI:** 10.1186/s13045-014-0068-2

**Published:** 2014-09-23

**Authors:** Yoshiaki Chinen, Natsumi Sakamoto, Hisao Nagoshi, Tomohiko Taki, Saori Maegawa, Shotaro Tatekawa, Taku Tsukamoto, Shinsuke Mizutani, Yuji Shimura, Mio Yamamoto-Sugitani, Tsutomu Kobayashi, Yosuke Matsumoto, Shigeo Horiike, Junya Kuroda, Masafumi Taniwaki

**Affiliations:** Division of Hematology and Oncology, Department of Medicine, Kyoto Prefectural University of Medicine, Graduate School of Medical Science, 465 Kajii-cho, Kamigyo-ku Kyoto, 602-8566 Japan; Department of Molecular Diagnostics and Therapeutics, Kyoto Prefectural University of Medicine, Graduate School of Medical Science, Kyoto, Japan

**Keywords:** Acute myeloid leukemia (AML), Long noncoding RNAs (lincRNAs), *PVT1*, *NSMCE2*, *CCDC26*

## Abstract

The pathogenetic roles of 8q24 amplified segments in leukemic cells with double minute chromosomes remain to be verified. Through comprehensive molecular analyses of 8q24 amplicons in leukemic cells from an acute myelogenous leukemia (AML) patient and AML-derived cell line HL60 cells, we identified two novel fusion genes between *NSMCE2* and long noncoding RNAs (lncRNAs), namely, *PVT1-NSMCE2* and *BF104016-NSMCE2*. Our study suggests that 8q24 amplicons are associated with the emergence of aberrant chimeric genes between *NSMCE2* and oncogenic lncRNAs, and also implicate that the chimeric genes involving lncRNAs potentially possess as-yet-unknown oncogenic functional roles.

**Electronic supplementary material:**

The online version of this article (doi:10.1186/s13045-014-0068-2) contains supplementary material, which is available to authorized users.

## To the Editor,

**Figure 1:**
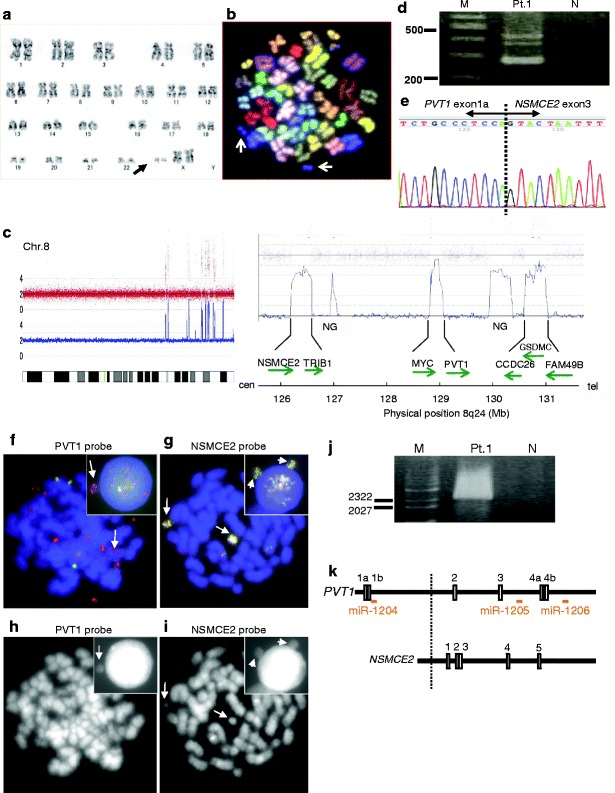
**Identification of** ***PVT1-NSMCE2*** **in the leukemic patient cells. (a)** G-banding analysis. Arrow indicates two marker chromosomes (mars). **(b)** SKY analysis for the patient identified two mars derived from chromosome 8 (arrows). **(c)** Copy number changes at 8q24 detected by high-resolution oligonucleotide array. *NSMCE2*, *TRIB1*, *MYC*, *PVT1*, *CCDC26, GSDMC*, and *FAM49B* are amplified. The direction of the arrows reflects the direction of gene transcription. NG: no gene. **(d)** Detection of three *PVT1-NSMCE2* fusion transcripts by RT-PCR. Primers were P1S and NSMCE2-Ex4AS for 5'-PVT1-NSMCE2-3'. Lane Pt.1: leukemic cells from the patient; lane N: water; lane M: size marker. **(e)** Sequence analysis of *NSMCE2* fusion transcript in the patient. **(f)** FISH finding of the patient using *PVT1* probe. Multiple red signals indicate extrachromosomal amplification of 5’*PVT1* on dmins. Co-localized red and green signals indicate normal *PVT1*. Inset shows 5’*PVT1* amplification in a micronucleus equivalent of mar (arrow). (Additional file 5: Figure S3) **(g)** FISH finding from the patient using an *NSMCE2* probe. Intense yellow signals indicate amplification of *NSMCE2* on mars and co-localized red and green signals signify normal *NSMCE2* on chromosome 8. Inset shows *NSMCE2* amplification in a micronucleus equivalent of mar (arrow). **(h** and **i)** DAPI pictures of metaphase cells corresponding to **(f)** and **(g)**. Arrows indicate mars. In metaphase, *NSMCE2* amplification was detectable on mars. 5’*PVT1* amplification were observed on dmins, however, *PVT1* FISH probe sets could not identify mars because of the background dmins (f and h). **(j)** Results of LDI-PCR. Primers were NSM38374 and NSM38666 for 5'-PVT1-NSMCE2-3'. Lane Pt.1: leukemic cells from the patient; lane N: water; lane M: size marker. (Additional file 4: Table S2) **(k)** Genomic mapping of *PVT1* and *NSMCE2* exons and breakpoint. White vertical boxes represent exons; dotted line represents breakpoint of *PVT1* and *NSMCE2* in the patient detected by LDI-PCR. Horizontal line indicates the location of miRNAs.

**Figure 2:**
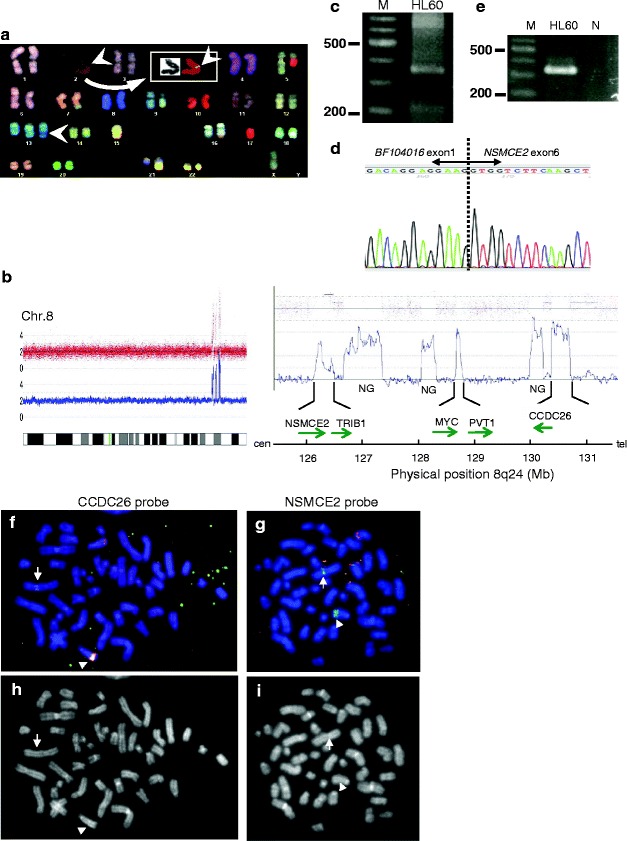
**Identification of** ***BF104016-NSMCE2*** **in HL60. (a)** Representative SKY karyotype of HL60 cells. Arrowheads indicate material inserted from chromosome 8 on ins(2;8) and der(13)hsr(8). Inset shows pseudocolor image of ins(2;8). **(b)** Copy number changes at 8q24 detected by high-resolution oligonucleotide array. *NSMCE2*, *TRIB1*, *MYC*, *PVT1* and *CCDC26* are amplified in HL60. The direction of the arrows reflects the direction of the gene transcription. NG: no gene. **(c)** Bubble PCR products detected by nested PCR using NVAMP1 and NSMCE2-Ex7AS for the first PCR, and NVAMP2 and NSM695 for the second. M: size marker. **(d)** Sequence analysis of *NSMCE2* fusion transcript of HL60. **(e)** Detection of *BF104016-NSMCE2* fusion transcripts by RT-PCR. Primers were BF104-1S and NSMCE2-Ex7AS for 5'-BF104016-NSMCE2-3'. Lanes N: water. (Additional file 4: Table S2) **(f)** FISH finding of HL60 using *CCDC26* probe. Intense co-localized red and green signal indicates amplification of the *CCDC26* gene on der(13)hsr(8) (arrowhead). A co-localized red and green signal is seen on ins(2;8) (arrow). Multiple green signals indicate amplification of the *5’CCDC26* gene on dmins. Co-localized red and green signals show normal *CCDC26*. **(g)** FISH finding of HL60 using *NSMCE2* probe. Intense green signals indicate amplification of 3’*NSMCE2* on der(13)hsr(8) (arrowhead) and ins(2;8) (arrow). Multiple red and green signals indicate amplification of the *NSMCE2* gene on dmins. Co-localized red and green signals indicate normal chromosomal *NSMCE2*. (Additional file 5: Figure S3) **(h** and **i)** DAPI pictures of metaphase cells corresponding to **(f)** and **(g)**. Arrow and arrowhead indicate ins(2;8) and der(13)hsr(8), respectively.

To gain insight into the role(s) of double minute chromosomes (dmins) in leukemia, we cytogenetically/molecularly analyzed 8q24 amplicons in patient-derived leukemic cells and AML-derived cell line (HL60) (See Additional file 1 for supplementary materials and methods). The patient was a 71-year-old female with AML (M2). The G-banding karyotype of leukemic cells was 47, XX, +mar [2]/48, XX, idem, +mar [6]/46, XX [7], containing two marker chromosomes (mars) from chromosome 8 (Figure 1a and b). DNA copy number analysis (CNA) revealed 13 high-level amplicons on 8q22.1-q24.2 (98.43 Mb-134.16 Mb) (Additional file 2: Table S1). SKY analysis of HL60 cells containing the 8q24 amplicons revealed that the representative karyotype was 44, X, der(5)t(5;17)(q11.2;q11.2), t(7;16;9)(q34;q24;p21), t(9;14)(q22;q22), +13, -15, -17, der(21)t(15;21)(q22;q21) [1]. CNA revealed several amplicons on 8q24.13-q24.12 (126.25 Mb-130.75 Mb) in the HL60 cells (Figure 2a and b). Consequently, three common amplicons were identified between 8q24.13-21 in the patient and the HL60 cells; i.e., the regions covering *NSMCE2* (8q24.13), *PVT1* (8q24.21) and *CCDC26* (8q24.21) (Figures 1c and 2b). Further investigation revealed three fusion transcripts between *PVT1* exon 1a and *NSMCE2* exon 3 in the patient (Figure 1d and e), and a fusion gene between exon 6 of *NSMCE2* and exon 1 of *BF104016*, a noncoding RNA sharing the sequence of *CCDC26* exon 4 (Additional file 3: Figure S1) (Additional file 4: Table S2), in the HL60 cells (Figure 2c-e). Both the *NSMCE2* and *PVT1* genes were amplified and located in a micronucleus in the patient (Figure 1f-i), and the genomic junction of 5’-*PVT1*-*NSMCE2*-3’ was located within intron 1 of *PVT1* and at 5’ upstream of exon 1 of *NSMCE2* (Figure 1j and k) (Additional file 5: Figure S3). In the HL60 cells, amplification of 3’*NSMCE2* and 5’*CCDC26* was co-localized on der(13)hsr(8), ins(2;8) and dmins (Figure 2e-h) (Additional file 5: Figure S3). Aberrant *NSMCE2* transcripts were higher than normal *NSMCE2* transcripts in the patient and the HL60 cells, while NSMCE2 protein expression did not correlate with normal or abnormal *NSMCE2* transcripts among the leukemic patient cells or the HL60 cells, suggesting the presence of regulatory mechanisms other than transcription (Additional file 6: Figure S2).

The present findings are consistent with previous studies demonstrating that segmental genome amplification of 8q24 contains recurrent *PVT1* fusion genes, which might be generated by chromothripsis [2,3]. Both lncRNAs, *PVT1* and *CCDC26,* harbor retroviral integration sites and are transcribed into multiple splice forms [4-6]. *PVT1* overexpression is induced by *MYC* or p53, contributing to suppression of apoptosis [7-9], whereas *PVT1* produces six annotated microRNAs that have been implicated in oncogenesis [3,10,11]. The chimeric transcripts involving *PVT1* may also regulate the expression of as-yet unspecified target genes through “enhancer-like functions” [12]. *CCDC26* amplification has been also identified as a recurrent abnormality that is associated with the response to retinoic acid-induced differentiation in AML [1,11,13-16]. This study is the first to identify *NSMCE2*-associated fusion genes in AML [17-19]. Knockdown of *NSMCE2* induces chromosomal instability and increases the frequency of chromosomal breakage and loss [20]. We speculate that *NSMCE2* gene rearrangement may potentially influence its function. Collectively, our study identified novel *PVT1-NSMCE2* and *CCDC26-NSMCE2* fusion genes that may play functional roles in leukemia.

## Additional files

Additional file 1 **Supplementary material information.**

Additional file 2: Table S1 CNAG analysis of the region between the *MTDH* and *LRRC6* genes on 8q24 in patient 1 with marker chromosomes. Results show the genomic size of the eight amplified segments that were selected based on the existence of known genes within them and their approximate positions.

Additional file 3: Figure S1 Association between *CCDC26* and *BF104016* at 8q24.21. The scale indicates the region 8q24.21. White boxes and grey boxes indicate exons of *CCDC26* and *BF104016* on the genetic locus at 8q24.21, respecitively. Vertical black lines indicate exons on the *CCDC26* isoform. According to the NCBI database, isoform 1 (BC070152.1) consists of four (1-2-3-4) exons, and isoform 2 (BC026098.1) consists of three (1a-3-4) exons. *BF104016* consists of 2 exons. The sequence of *BF104016* exon 2 is partly consistent with that of *CCDC26* exon 4. ORF: hypothetical open reading frame.

Additional file 4: Table S2 Sequences of the primers used in this study.

Additional file 5: Figure S3 Identification of breakpoints region at 8q24 by FISH. Upper panel: location of FISH probes shown as color bars and position of *NSMCE2*, *TRIB1*, *MYC*, and *PVT1* genes at 8q24. Vertical black lines indicate exons of *NSMCE2*, *PVT1*, and *BF104016*. Lower panel: mapping of breakpoint in leukemic cells of patient 1 and HL60. Gray boxes indicate amplified regions detected.

Additional file 6: Figure S2 Expression of *NSMCE2* in patient 1 and AML-derived cell lines. (a) *NSMCE2* mRNA levels measured by RQ-PCR (n=3, mean ± SD). Theoretically, the NSMCE2 7-8 primer/probe can amplify both normal and aberrant *NSMCE2* transcripts, while the NSMCE2 2-3 primer/probe set which can amplify only normal *NSMCE2* transcript. *NSMCE2* mRNA levels were normalized to β-actin and are relative to the control mRNA extracted from normal BM cells. *NSMCE2* mRNA levels amplified by the NSMCE2 7-8 primer/probe set are higher than those amplified by the NSMCE2 2-3 primer/probe set in patient 1, HL60 and KG1 cells. (b) Protein analysis using the anti-NSMCE2 antibody in cells. Blot for β-actin was used as loading control. Lane 1: normal BM; lane 2: KG1; lane 3: HL60. (c and d) IHC analysis of NSMCE2 expression in BM of patient 1 (c) and normal BM (d). NSMCE2 expression of leukemic cells was not higher than that of normal BM cells. Monocytes and megakaryocytes showed strong positive signals in their cytoplasm.

## Abbreviations

dmins: Double minute chromosomes
hsr: Homogeneously staining regions
FISH: Fluorescence *in situ* hybridization
lncRNAs: Long noncoding RNAs
AML: Acute myeloid leukemia
MDS: Myelodysplastic syndromes
NSMCE2: Non-SMC element 2
SKY: Spectral karyotyping
RT-PCR: Reverse transcription-polymerase chain reaction
LDI-PCR: Long-distance inverse PCR
