# Supplementary material for: 8q24 amplified segments involve novel fusion genes between NSMCE2 and long noncoding RNAs in acute myelogenous leukemia
Source: J Hematol Oncol. 2014 Sep 23;7:68. doi: 10.1186/s13045-014-0068-2 (PMC4176872; doi:10.1186/s13045-014-0068-2)
Supplement: Supplementary file 1 — Supplementary material information. [file 13045_2014_68_MOESM1_ESM.docx]

**Supplementary Material Information**

**Supplementary Methods**

**Materials and Methods**

**Cells and reagents.** Cells were maintained in RPMI-1640 containing 10% fetal calf serum, 2 mM L-glutamate, and penicillin/streptomycin. AML-derived cell lines, HL60 and KG1, were obtained from the American type culture collection (ATCC, Manassas, VA). The use of the clinical sample including a healthy volunteer was approved by the Institutional Review Board of Kyoto Prefectural University of Medicine (Kyoto, Japan), and the written informed consent was obtained from the patient along with the declaration of Helsinki.

**Spectral karyotyping (SKY) analysis.** SKY analysis was performed with a Sky painting kit (Applied Spectral Imaging, MigdalHa'Emek, Israel). Karyotype was described according to the International System for Human Cytogenetic Nomenclature (ISCN, 2013).

**Genome copy number and breakpoint analysis using high-resolution oligonucleotide array.** The DNA gain and loss assay on the basis of high-density oligonucleotide microarrays (GeneChip Human Mapping 50K, 250K, or 6.0 SNP array, Affymetrix) was conducted with genomic DNA. Breakpoints were identified by the means of genome copy number analysis, and the SNP array data were analyzed to determine total copy numbers using the CNAG3.0 or 3.3 programs.

**Reverse transcription-polymerase chain reaction (RT-PCR) and sequencing analysis.** For detection of the *PVT1-NSMCE2* fusion gene, the following primers were used: P1S (forward primer in exon 1a of *PVT1*), and NSMCE2-Ex4AS (reverse primer in exon 4 of *NSMCE2*), and for detection of the *BF104016-NSMCE2* fusion gene, BF104-1S (forward primer in *BF104016*), and NSMCE2-Ex7AS (reverse primer in exon 7 of *NSMCE2*) (Supplementary Table 2). The *PVT1* mRNA sequence was obtained from NCBI using the accession nos. M34428, M34429, and NR_003367.

**Bubble PCR for cDNA.** Bubble PCR and nested PCR were performed using the primers NVAMP1 (bubble oligo) and NSMCE2-Ex7AS for the first-round PCR, and NVAMP2 (bubble oligo) and NSM695 for the nested PCR. Poly(A)+ RNA was extracted from the patient’s leukemic cells with the QuickPrep Micro mRNA Purification Kit (GE Healthcare, Waukesha, WI). Double-stranded cDNAs were synthesized, digested with both *EcoR*V and *Hinc*II, and ligated with bubble oligo. The sequences of all primers used in this study are listed in Supplementary Table S2.

**Fluorescence *in situ* hybridization** (**FISH) analysis.** The *PVT1* probe set to identify *PVT1* rearrangements consisted of two BAC clones comprising CTD-2267H22, a fragment approximately 120 kb in length covering 5’ regions of *PVT1*, and RP11-164J24, a fragment approximately 190 kb in length covering 3’ regions of *PVT1*. To identify *NSMCE2* rearrangements, we prepared specific probe set (*NSMCE2* probe) using two BAC clones, RP11-313A10 and RP11-550A5, encompassing the 5’- and the 3’-half of *NSMCE2*. To identify *CCDC26* rearrangements, we prepared specific probe set (*CCDC26* probe) using two BAC clones RP11-26E5, a fragment approximately 180 kb in length covering 3’ regions of *CCDC26*, and RP11-259L23, a fragment approximately 170 kb in length covering and 5’ regions of *CCDC26*. Genomic locations of FISH probes are demonstrated in Supplementary Figure S3.

**Long-distance inverse (LDI)-PCR and nucleotide sequencing.** Briefly, 2.5 μg of genomic DNA was digested with *Bgl*II. The digested and purified DNA was then diluted to 1 μg/mL and 300 ng was self-ligated using the DNA Ligation Kits (Takara, Kyoto, Japan). After purification, long-distance PCR was performed using the KOD FX enzyme (Toyobo, Osaka, Japan). Reaction conditions were: denaturation at 94°C for 2min followed by 35 cycles of denaturation at 94°C for 10sec, annealing at 63°C for 30 sec, and extension for 210 sec at 68°C. The sequences of the primers used are listed in Supplementary Table S2. The PCR product was then subjected to sequence analysis. Sequences of the regions of interest were analyzed with the aid of the University of California Santa Cruz Genome Bioinformatics database and using the Basic Local Alignment Search Tool (BLAST). If necessary, subcloned PCR products were analyzed.

**Real-time Quantitative-PCR (RQ-PCR).** *NSMCE2* mRNA levels were determined with primer/probe sets from ABI Assays-on-Demand (Applied Biosystems) using the ABI Prism 7300 system (Applied Biosystems). Each mRNA level was normalized to that of *β-actin* (Taqman β-actin control reagents). Primers/probe sets used were Assays-on-Demand NSMCE2 2-3 (Hs01075591_g1) and NSMCE2 7-8 (Hs00329126_m1), which were used to distinguish normal and abnormal chimeric *NMSCE2* transcripts from normal *NSMCE2*, since the NSMCE2 2-3 probe spans the 5'UTR region of *NSMCE2*, and NSMCE2 7-8 can detect all mRNAs including normal and fusion transcripts. In addition to a patient’s cells, normal BM cells, HL60 and KG1 were analyzed. A primers/probe set specific for *PVT1* was Assays-on-Demand PVT1 (Hs01069023_m1). Each assay was done in triplicate. *NSMCE2*/*β-actin* ratios were expressed relative to those for normal BM cells to compare the expression levels of *NSMCE2* transcripts between leukemic cells and normal BM cells.

**Western blot analysis and immunohistochemistory (IHC).** The primary antibodies used were ab105363 (Abcam plc, Cambridge, UK), which can detect a 15-amino acid peptide near the C-terminus of human NSMCE2, and β-Actin (Sigma, St Louis, MO). Immunohistochemical staining was performed with antibodies specific for NSMCE2 (NBP1-76263, Novus Biologicals, Littleton, CO).

**Supplementary Legends**

**Table S1. CNAG analysis of the region between the *MTDH* and *LRRC6* genes on 8q24 in patient 1 with marker chromosomes.** Results show the genomic size of the eight amplified segments that were selected based on the existence of known genes within them and their approximate positions.

**Table S2. Sequences of the primers used in this study.**

**Figure S1. Association between *CCDC26* and *BF104016* at 8q24.21.** The scale indicates the region 8q24.21. White boxes and grey boxes indicate exons of *CCDC26* and *BF104016* on the genetic locus at 8q24.21, respecitively. Vertical black lines indicate exons on the *CCDC26* isoform. According to the NCBI database, isoform 1 (BC070152.1) consists of four (1-2-3-4) exons, and isoform 2 (BC026098.1) consists of three (1a-3-4) exons. *BF104016* consists of 2 exons. The sequence of *BF104016* exon 2 is partly consistent with that of *CCDC26* exon 4. ORF: hypothetical open reading frame.

**Figure S2. Expression of *NSMCE2* in patient 1 and AML-derived cell lines.** (a) *NSMCE2* mRNA levels measured by RQ-PCR (n=3, mean ± SD). Theoretically, the NSMCE2 7-8 primer/probe can amplify both normal and aberrant *NSMCE2* transcripts, while the NSMCE2 2-3 primer/probe set which can amplify only normal *NSMCE2* transcript. *NSMCE2* mRNA levels were normalized to β-actin and are relative to the control mRNA extracted from normal BM cells. *NSMCE2* mRNA levels amplified by the NSMCE2 7-8 primer/probe set are higher than those amplified by the NSMCE2 2-3 primer/probe set in patient 1, HL60 and KG1 cells. (b) Protein analysis using the anti-NSMCE2 antibody in cells. Blot for β-actin was used as loading control. Lane 1: normal BM; lane 2: KG1; lane 3: HL60. (c and d) IHC analysis of NSMCE2 expression in BM of patient 1 (c) and normal BM (d). NSMCE2 expression of leukemic cells was not higher than that of normal BM cells. Monocytes and megakaryocytes showed strong positive signals in their cytoplasm.

**Figure S3. Identification of breakpoints region at 8q24 by FISH.** Upper panel: location of FISH probes shown as color bars and position of *NSMCE2*, *TRIB1*, *MYC*, and *PVT1* genes at 8q24. Vertical black lines indicate exons of *NSMCE2*, *PVT1*, and *BF104016*. Lower panel: mapping of breakpoint in leukemic cells of patient 1 and HL60. Gray boxes indicate amplified regions detected.
