# Supplementary material for: 8q24 amplified segments involve novel fusion genes between NSMCE2 and long noncoding RNAs in acute myelogenous leukemia
Source: J Hematol Oncol. 2014 Sep 23;7:68. doi: 10.1186/s13045-014-0068-2 (PMC4176872; doi:10.1186/s13045-014-0068-2)
Supplement: Supplementary file 2 — CNAG analysis of the region between the MTDH and LRRC6 genes on 8q24 in patient 1 with marker chromosomes. Results show the genomic size of the eight amplified segments that were selected based on the existence of known genes within them and their approximate positions. [file 13045_2014_68_MOESM2_ESM.pptx]

## Slide 1
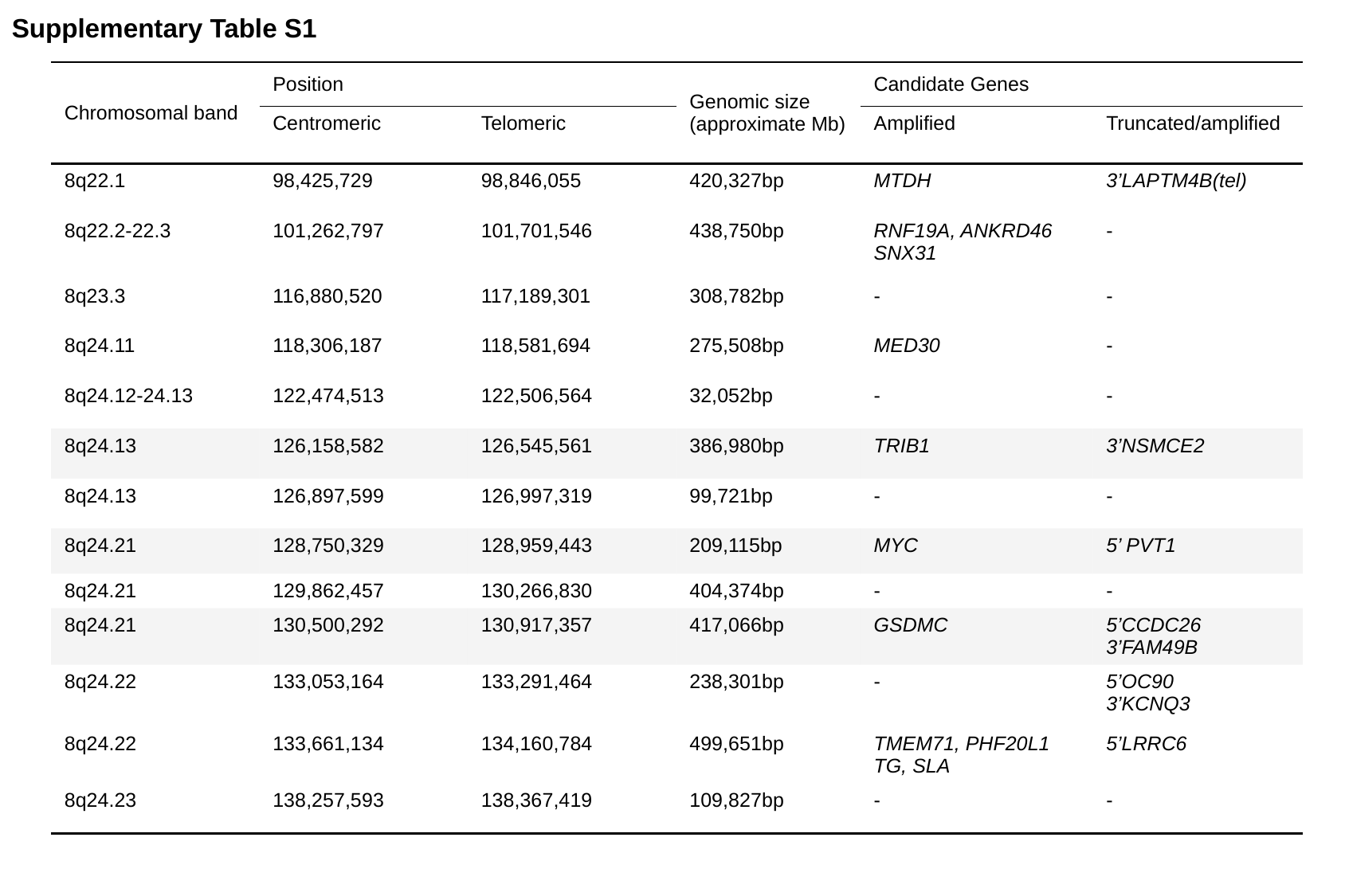

Supplementary Table S1
| Chromosomal band | Position | | Genomic size (approximate Mb) | Candidate Genes | |
| --- | --- | --- | --- | --- | --- |
| | Centromeric | Telomeric | | Amplified | Truncated/amplified |
| 8q22.1 | 98,425,729 | 98,846,055 | 420,327bp | MTDH | 3’LAPTM4B(tel) |
| 8q22.2-22.3 | 101,262,797 | 101,701,546 | 438,750bp | RNF19A, ANKRD46 SNX31 | - |
| 8q23.3 | 116,880,520 | 117,189,301 | 308,782bp | - | - |
| 8q24.11 | 118,306,187 | 118,581,694 | 275,508bp | MED30 | - |
| 8q24.12-24.13 | 122,474,513 | 122,506,564 | 32,052bp | - | - |
| 8q24.13 | 126,158,582 | 126,545,561 | 386,980bp | TRIB1 | 3’NSMCE2 |
| 8q24.13 | 126,897,599 | 126,997,319 | 99,721bp | - | - |
| 8q24.21 | 128,750,329 | 128,959,443 | 209,115bp | MYC | 5’ PVT1 |
| 8q24.21 | 129,862,457 | 130,266,830 | 404,374bp | - | - |
| 8q24.21 | 130,500,292 | 130,917,357 | 417,066bp | GSDMC | 5’CCDC26 3’FAM49B |
| 8q24.22 | 133,053,164 | 133,291,464 | 238,301bp | - | 5’OC90 3’KCNQ3 |
| 8q24.22 | 133,661,134 | 134,160,784 | 499,651bp | TMEM71, PHF20L1 TG, SLA | 5’LRRC6 |
| 8q24.23 | 138,257,593 | 138,367,419 | 109,827bp | - | - |
