# Supplementary material for: 8q24 amplified segments involve novel fusion genes between NSMCE2 and long noncoding RNAs in acute myelogenous leukemia
Source: J Hematol Oncol. 2014 Sep 23;7:68. doi: 10.1186/s13045-014-0068-2 (PMC4176872; doi:10.1186/s13045-014-0068-2)
Supplement: Supplementary file 3 — Association between CCDC26 and BF104016 at 8q24.21. The scale indicates the region 8q24.21. White boxes and grey boxes indicate exons of CCDC26 and BF104016 on the genetic locus at 8q24.21, respecitively. Vertical black lines indicate exons on the CCDC26 isoform. According to the NCBI database, isoform 1 (BC070152.1) consists of four (1-2-3-4) exons, and isoform 2 (BC026098.1) consists of three (1a-3-4) exons. BF104016 consists of 2 exons. The sequence of BF104016 exon 2 is partly consistent with that of CCDC26 exon 4. ORF: hypothetical open reading frame. [file 13045_2014_68_MOESM3_ESM.pptx]

## Slide 1
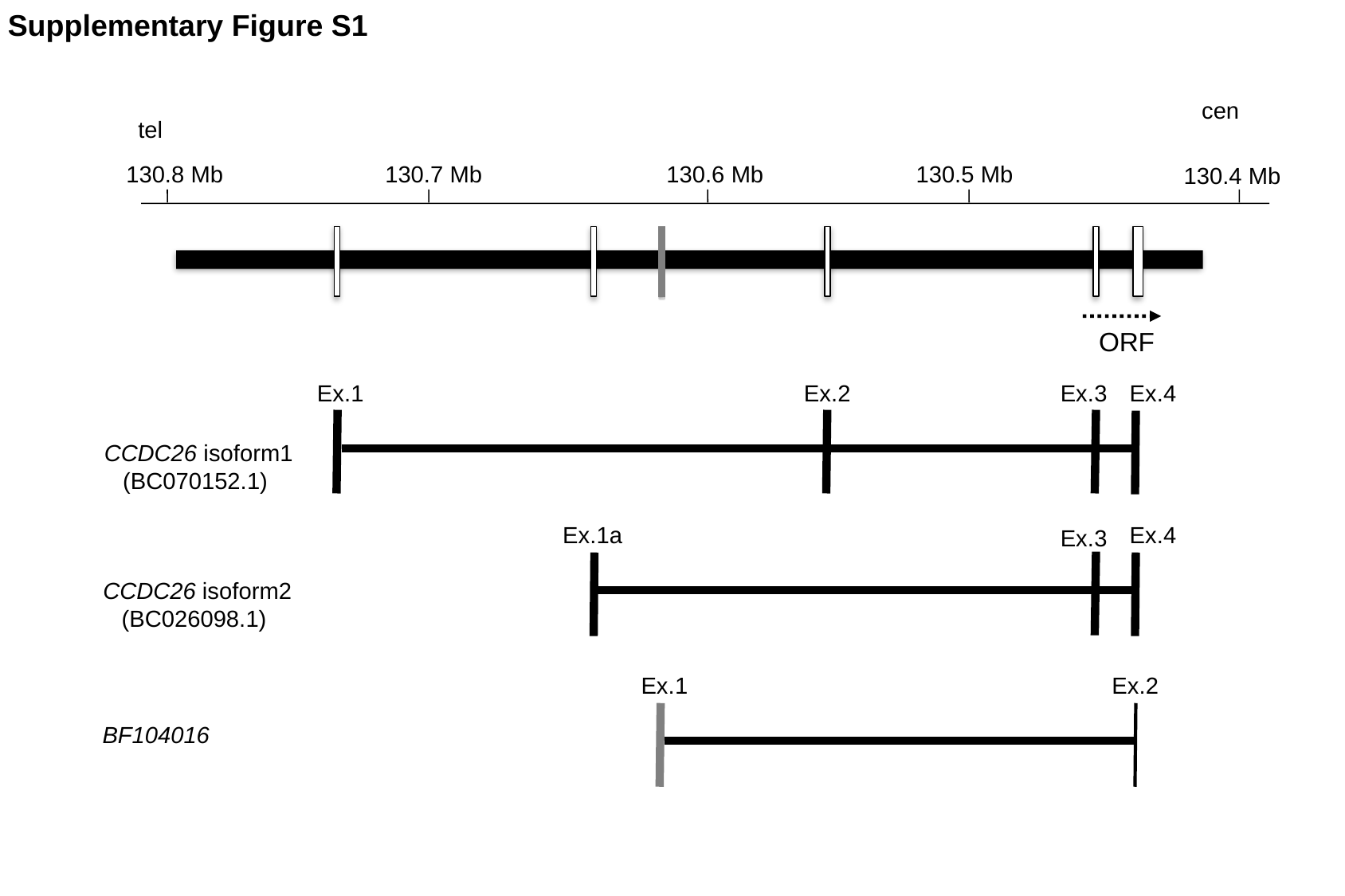

Supplementary Figure S1
cen
tel
130.7 Mb
130.8 Mb
130.6 Mb
130.5 Mb
130.4 Mb
ORF
Ex.1
Ex.2
Ex.3
Ex.4
CCDC26 isoform1 (BC070152.1)
Ex.1a
Ex.4
Ex.3
CCDC26 isoform2 (BC026098.1)
Ex.1
Ex.2
BF104016
