# Supplementary material for: 8q24 amplified segments involve novel fusion genes between NSMCE2 and long noncoding RNAs in acute myelogenous leukemia
Source: J Hematol Oncol. 2014 Sep 23;7:68. doi: 10.1186/s13045-014-0068-2 (PMC4176872; doi:10.1186/s13045-014-0068-2)
Supplement: Supplementary file 4 — Sequences of the primers used in this study. [file 13045_2014_68_MOESM4_ESM.pptx]

## Slide 1
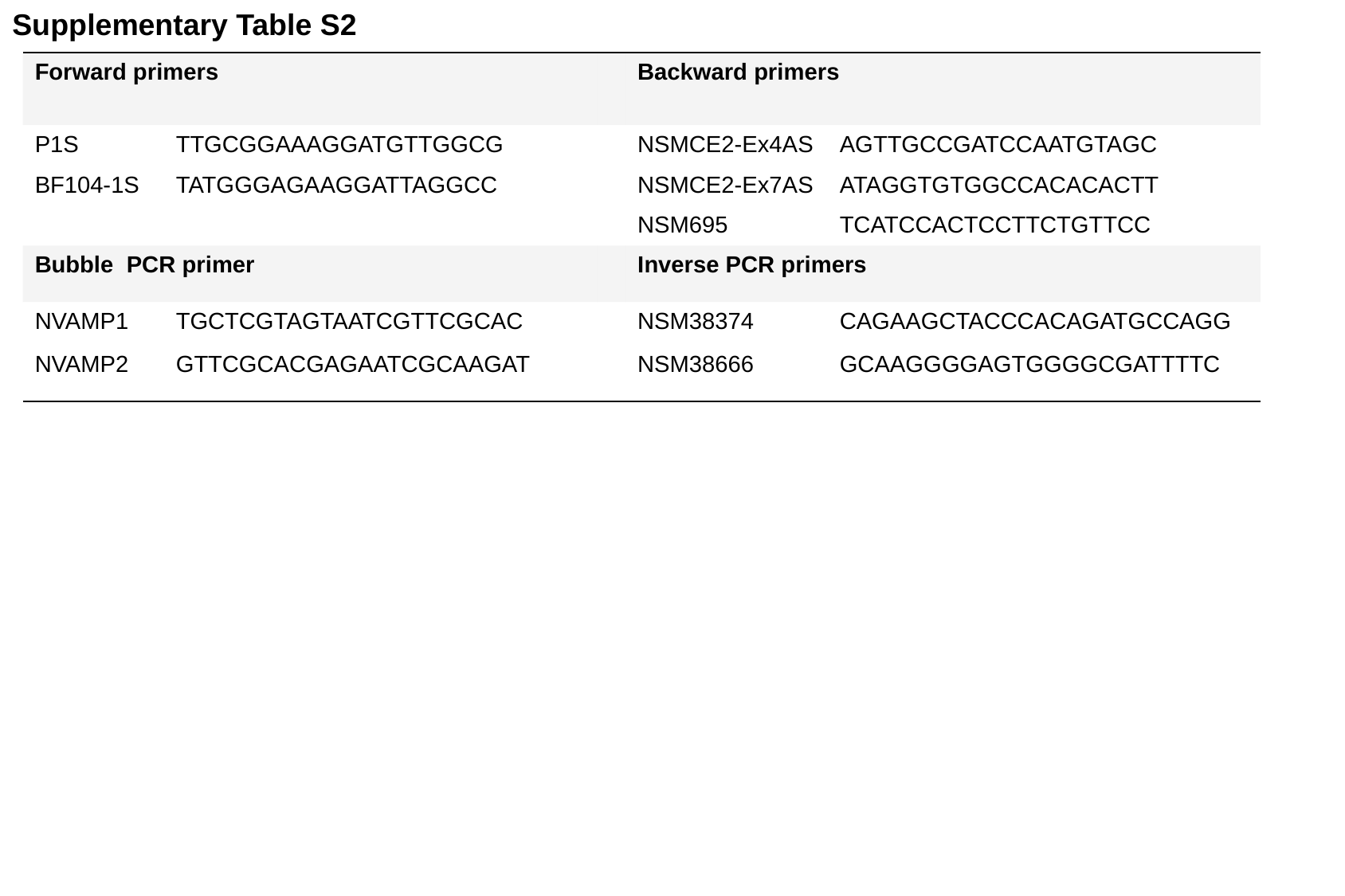

Supplementary Table S2
| Forward primers | | | Backward primers | |
| --- | --- | --- | --- | --- |
| P1S | TTGCGGAAAGGATGTTGGCG | | NSMCE2-Ex4AS | AGTTGCCGATCCAATGTAGC |
| BF104-1S | TATGGGAGAAGGATTAGGCC | | NSMCE2-Ex7AS | ATAGGTGTGGCCACACACTT |
| | | | NSM695 | TCATCCACTCCTTCTGTTCC |
| Bubble PCR primer | | | Inverse PCR primers | |
| NVAMP1 | TGCTCGTAGTAATCGTTCGCAC | | NSM38374 | CAGAAGCTACCCACAGATGCCAGG |
| NVAMP2 | GTTCGCACGAGAATCGCAAGAT | | NSM38666 | GCAAGGGGAGTGGGGCGATTTTC |
