# Supplementary material for: 8q24 amplified segments involve novel fusion genes between NSMCE2 and long noncoding RNAs in acute myelogenous leukemia
Source: J Hematol Oncol. 2014 Sep 23;7:68. doi: 10.1186/s13045-014-0068-2 (PMC4176872; doi:10.1186/s13045-014-0068-2)
Supplement: Supplementary file 5 — Identification of breakpoints region at 8q24 by FISH. Upper panel: location of FISH probes shown as color bars and position of NSMCE2, TRIB1, MYC, and PVT1 genes at 8q24. Vertical black lines indicate exons of NSMCE2, PVT1, and BF104016. Lower panel: mapping of breakpoint in leukemic cells of patient 1 and HL60. Gray boxes indicate amplified regions detected. [file 13045_2014_68_MOESM5_ESM.pptx]

## Slide 1
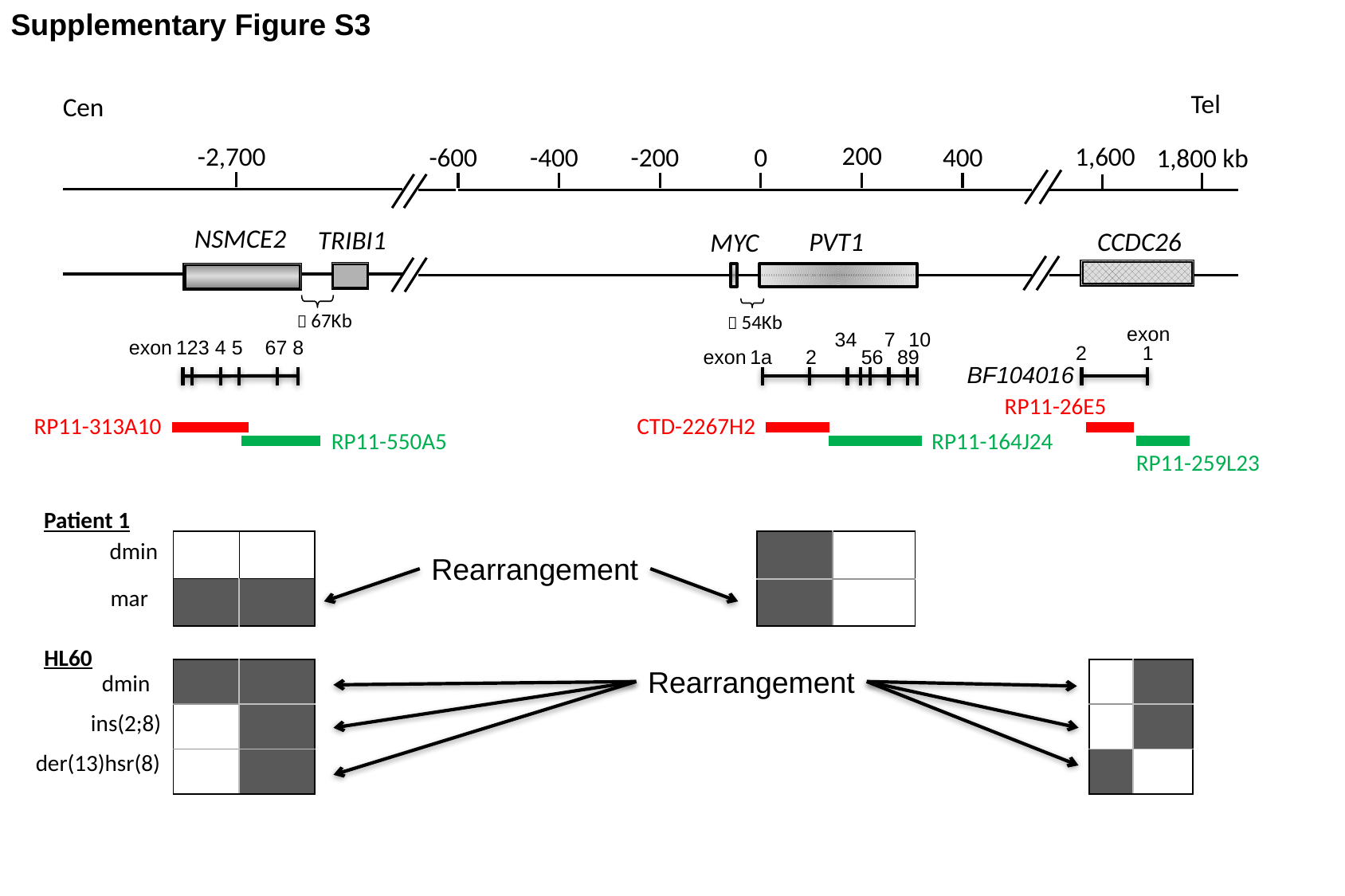

Supplementary Figure S3
Tel
Cen
200
-2,700
1,600
-600
-400
-200
0
400
1,800 kb
NSMCE2
TRIBI1
PVT1
CCDC26
MYC
～67Kb
～54Kb
exon
10
34
7
89
exon
123 4 5 67 8
 2 1
exon
1a 2 56
BF104016
RP11-26E5
RP11-313A10
CTD-2267H2
RP11-550A5
RP11-164J24
RP11-259L23
Patient 1
dmin
| | | | | | | | | | |
| --- | --- | --- | --- | --- | --- | --- | --- | --- | --- |
| | | | | | | | | | |
Rearrangement
mar
HL60
dmin
Rearrangement
| | | | | | | | | | |
| --- | --- | --- | --- | --- | --- | --- | --- | --- | --- |
| | | | | | | | | | |
| | | | | | | | | | |
| | |
| --- | --- |
| | |
| | |
ins(2;8)
der(13)hsr(8)
