# Supplementary material for: 8q24 amplified segments involve novel fusion genes between NSMCE2 and long noncoding RNAs in acute myelogenous leukemia
Source: J Hematol Oncol. 2014 Sep 23;7:68. doi: 10.1186/s13045-014-0068-2 (PMC4176872; doi:10.1186/s13045-014-0068-2)
Supplement: Supplementary file 6 — Expression of NSMCE2 in patient 1 and AML-derived cell lines. (a) NSMCE2 mRNA levels measured by RQ-PCR (n=3, mean ± SD). Theoretically, the NSMCE2 7-8 primer/probe can amplify both normal and aberrant NSMCE2 transcripts, while the NSMCE2 2-3 primer/probe set which can amplify only normal NSMCE2 transcript. NSMCE2 mRNA levels were normalized to β-actin and are relative to the control mRNA extracted from normal BM cells. NSMCE2 mRNA levels amplified by the NSMCE2 7-8 primer/probe set are higher than those amplified by the NSMCE2 2-3 primer/probe set in patient 1, HL60 and KG1 cells. (b) Protein analysis using the anti-NSMCE2 antibody in cells. Blot for β-actin was used as loading control. Lane 1: normal BM; lane 2: KG1; lane 3: HL60. (c and d) IHC analysis of NSMCE2 expression in BM of patient 1 (c) and normal BM (d). NSMCE2 expression of leukemic cells was not higher than that of normal BM cells. Monocytes and megakaryocytes showed strong positive signals in their cytoplasm. [file 13045_2014_68_MOESM6_ESM.pptx]

## Slide 1
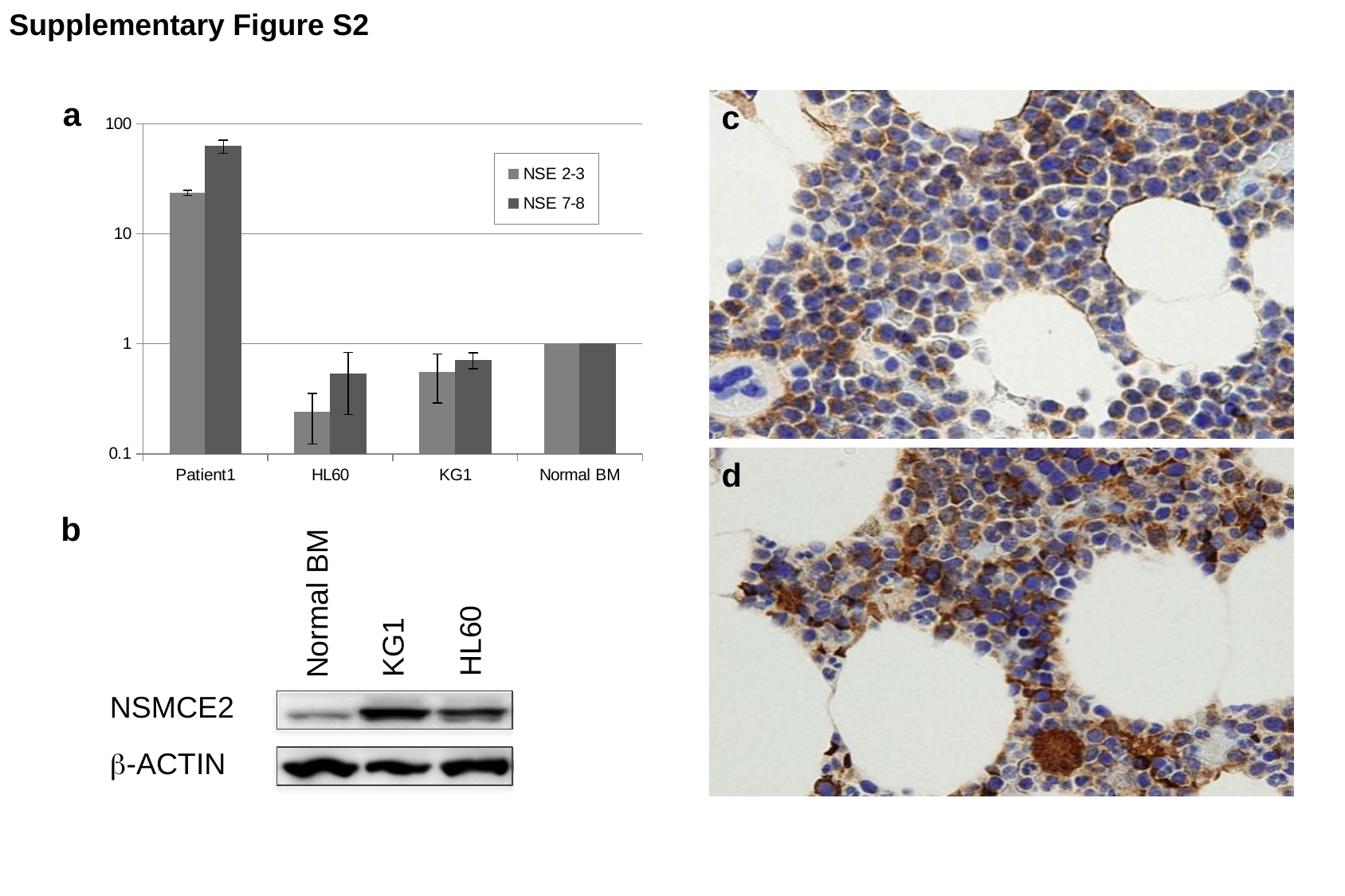

Supplementary Figure S2
a
c
### Chart
| Category | NSE 2-3 | NSE 7-8 |
|---|---|---|
| Patient1 | 23.57339524109586 | 62.633531726363 |
| HL60 | 0.237776562223245 | 0.530124607547836 |
| KG1 | 0.549576980517502 | 0.71170593801787 |
| Normal BM | 1.0 | 1.0 |
d
b
HL60
KG1
Normal BM
NSMCE2
b-ACTIN
